# Potential of Pseudoshikonin I Isolated from Lithospermi Radix as Inhibitors of MMPs in IL-1β-Induced SW1353 Cells

**DOI:** 10.3390/ijms17081350

**Published:** 2016-08-18

**Authors:** Dae Young Lee, Soo-Im Choi, Se Hee Han, Ye-Joo Lee, Jong-Gil Choi, Young-Seob Lee, Je Hun Choi, Seung-Eun Lee, Geum-Soog Kim

**Affiliations:** 1 Department of Herbal Crop Research, National Institute of Horticultural and Herbal Science, RDA, Eumseong 27709, Korea; dylee0809@gmail.com (D.Y.L.); yejoo@rda.go.kr (Y.-J.L.); youngseoblee@korea.kr (Y.-S.L.); jehun@rda.go.kr (J.H.C.); seungeun@rda.go.kr (S.-E.L.); 2 YD Life Science Research Institutes, YD Life Science Co., Ltd., Seongnam 13235, Korea; sh.han@ydgls.com (S.-H.H.); Jonggil@rda.go.kr (J.-G.C.); 3 Department of Food Science & Biotechnology, Chungbuk National University, Cheongju 28644, Korea

**Keywords:** Lithospermi radix, nuclear magnetic resonance (NMR), pseudoshikonin I, matrix-metalloproteinase (MMPs)

## Abstract

Pseudoshikonin I, the new bioactive constituent of Lithospermi radix, was isolated from this methanol extract by employing reverse-phase medium-pressure liquid chromatography (MPLC) using acetonitrile/water solvent system as eluents. The chemical structure was determined based on spectroscopic techniques, including 1D NMR (^1^H, ^13^C, DEPT), 2D NMR (gCOSY, gHMBC, gHMQC), and QTOF/MS data. In this study, we demonstrated the effect of pseudoshikonin I on matrix-metalloproteinase (MMPs) activation and expression in interleukin (IL)-1β-induced SW1353 chondrosarcoma cells. MMPs are considered important for the maintenance of the extracellular matrix. Following treatment with PS, active MMP-1, -2, -3, -9, -13 and TIMP-2 were quantified in the SW1353 cell culture supernatants using a commercially available ELISA kit. The mRNA expression of MMPs in SW1353 cells was measured by RT-PCR. Pseudoshikonin I treatment effectively protected the activation on all tested MMPs in a dose-dependent manner. TIMP-2 mRNA expression was significantly upregulated by pseudoshikonin I treatment. Overall, we elucidated the inhibitory effect of pseudoshikonin on MMPs, and we suggest its use as a potential novel anti-osteoarthritis agent.

## 1. Introduction

*Lithospermum erythrorhizon* Sieb. et Zucc., called “Jichi” in Korea and “Zicao” in China, is a perennial herbaceous plant and has been used as traditional herbal medicine and as natural dye for staining fabrics and food colorants, mainly in Korea, Japan, and China [1]. It has been traditionally used for the improvement of vascular circulation, removal of fever, detoxification, wound healing, and treatment of hematemesis, hematuria, constipation, eczema, and urinary tract infection [2]. In particular, it has been shown to possess many diverse activities, including anticancer [3,4], antioxidant [5,6,7,8], anti-inflammatory [9,10], antibacterial [11], antifungal [12], hepatoprotective [13], neuroprotective [14], and cosmetic [15] effects. The bioactive phytochemicals of this herb are naphthoquinone compounds, such as shikonin, and its derivatives, such as acetylshikonin, alkannan, isobutyl-shikonin, β,β-dimehtyl-acrylshikonin, β-hydroxyl isovaloryl shikonin, and tetracrylshikonin [6,7,16]. Our previous study showed that the supercritical fluid extract and napthoquinone compounds, like shikonin and acetylshikonin, from Lithospermi radix have strong protective activities on chondrocytes and MIA-induced osteoarthritis in rats [17].

This study sought to evaluate the molecular structure and cartilage protection activities of a new compound from the Lithospermi radix through the inhibitory effect on matrix-metalloproteinase (MMPs) activation and expression in interleukin-1β-induced SW1353 chondrosarcoma cells.

## 2. Results and Discussion

A crude methanol extract of Lithspermi radix was purified by preparative MPLC to yield a new compound, pseudoshikonin I.

Compound **1** was isolated as reddish powder (methanol), and its TLC had a deep purple color after spraying with 10% H_2_SO_4_ and heating. The molecular formula was established to be C_21_H_24_O_4_ from the molecular ion peak *m*/*z* 339.12884 [M − H]^−^ in the negative QTOF/MS.

**Figure 1:**
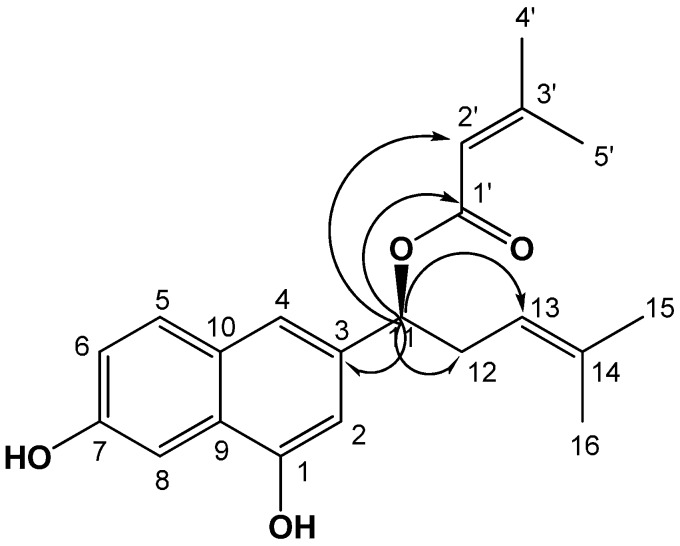
Chemical structure of compound **1** isolated from the Lithospermi radix and key gHMBC (**arrow**) correlations of compound **1**.

The full assignment of the ^1^H and ^13^C NMR signals of compound **1**, secured by gCOSY, gHSQC, and gHMBC spectra, and the comparison of these data with those of shikonin [7] and β,β-dimethylacrylshikonin [18] indicated the similarities of this compound. The significant difference of compound was the presence of OH signals in compound **1**, instead of two carbonyl signals in shikonin. The ^1^H-NMR spectrum showed two olefinic methine proton signals (δ_H_ 7.42 (1H, s, H-2), 6.95 (1H, s, H-4)) and three olefin methine proton signals (δ_H_ 7.14 (1H, d, *J* = 3.2 Hz, H-8), 6.69 (1H, d, *J* = 8.8 Hz, H-5), 6.54 (1H, dd, *J* = 8.8, 3.2 Hz, H-6)); thus, suggesting that compound **1** has a meta-coupled aromatic, including a 1,2,4-3-substitute aromatic ring in the naphthalene moiety. The ^1^H and ^13^C NMR spectra of compound **1** showed characteristic signals for 4-methylpent-3-enyl side chain (δ_H_ 5.75 (H-11), 5.11 (H-13), 2.55 (H-12), 1.74 (H-16), 1.56 (H-15); δ_C_ 119.5 (C-14), 109.7 (C-13), 69.3 (C-11), 34.8 (C-12), 20.3 (C-15), 18.0 (C-16)) and 3-methylbut-2-enoate side chain (δ_H_ 5.69 (H-2′), 2.14 (H-5′), 1.89 (H-4′); δ_C_ 167.5 (C-1′), 139.3 (C-2′), 135.6 (C-3′), 27.4 (C-5′), 25.9 (C-4′)), which were also present in shikonin and β,β-dimethylacrylshikonin. In the gHMBC spectrum, long-range correlation was shown between the oxgenated methine proton signal (δ_H_ 5.75 (H-11)) and carboxyl carbon signal (δ_C_ 167.5 (C-1′)), with the olefine methine carbon signal (δ_C_ 139.3 (C-2′)) of the butyl group indicating that the butyl group was linked to the hydroxyl of C-11. In addition, correlations were observed between the oxgenated methine proton signal (H-11) and the quaternary carbon signal (δ_C_ 128.4 (C-3)) and between methylene carbon signal (δ_c_ 34.8 (C-12)) and olefinic methine carbon signal (δ_c_ 109.7 (C-13)) (Figure 1). The absolute configurations of compound **1** were resolved using optical rotation data. The comparison of the specific rotation of compound **1** ([α]${}_{D}^{25}$ +75.0; *c* 0.10, CHCl_3_) with alkannin ([α]${}_{D}^{20}$ −150.5; *c* 0.05, benzene) and shikonin ([α]${}_{D}^{20}$ +135.6; *c* 0.05, benzene) [19] was consistent with compound **1** possessing an 11*S* absolute configuration. Based on the data above, the chemical structure of compound **1** was determined to be 1-(1,7-dihydroxynaphthalen-3-yl)-4-methylpent-3-enyl 3-methylbut-2-enoate named pseudoshikonin I.

### 2.1. Cell Viability of Pseudoshikonin I on SW1353 Cells

**Figure 2:**
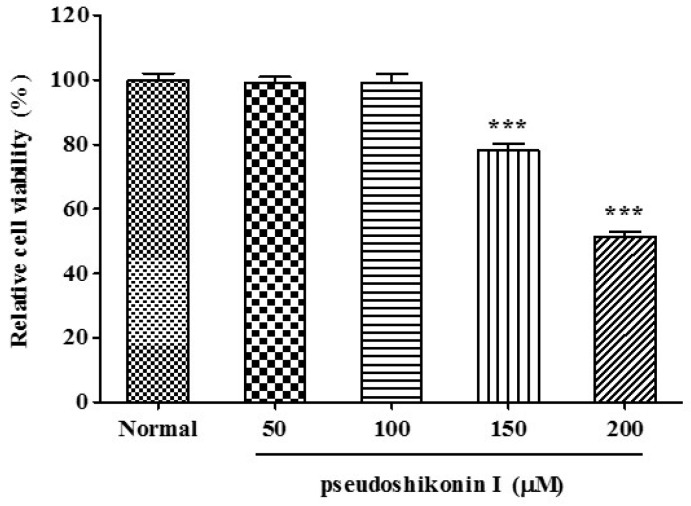
Cell viability of pseudoshikonin I in SW1353 cells. SW1353 cells were treated with indicated concentrations of pseudoshikonin I for 24 h. Normal represents samples only supplied with DMEM and cell viability assay being conducted by the MTT method. Results are expressed as the percentage change of control condition in which cells were grown in medium without pseudoshikonin I. Results shown are mean S.D. of experiments in triplicate. * *p* < 0.05; ** *p* < 0.01; *** *p* < 0.001 compared with control.

Following incubation with pseudoshikonin I for 24 h, no significant difference was shown between the viability of cells in the control and that in the 10–100 μM of PSI treatment. As shown in Figure 2; however, more than 100 μM of pseudoshikonin I treatment significantly inhibited the proliferation of SW1353 cells. The IC_50_ value was 201.5 μM of pseudoshikonin I on SW1353 cells.

### 2.2. Effect of Pseudoshikonin I on MMPs Activity in IL-1β-Induced SW1353 Cells

MMP-1, -2, -3, -9, -13, and TIMP-2 productions in IL-1β-stimulated SW1353 cells in the absence or presence of pseudoshikonin I were profiled. IL-1β contributes to the elevated expression of MMPs by chondrocytes in vitro and in vivo [20,21]. Previous studies demonstrated the expression and activation of MMP-1, MMP-2, MMP-3, MMP-9, and MMP-13 in IL-1β-stimulated human chondrosarcoma cells [22].

Cells were co-treated with pseudoshikonin I and IL-1β (20 ng/mL) for 24 h. MMP concentrations in the cell supernatants were expressed as ng total MMP-13/10^6^ to standardize the amounts between cultures. As a result (Table 1), in the presence of IL-1β (control group), MMP-13 production increased (*p* < 0.01), with concentrations reaching 4.97 ± 0.50 ng/10^6^ cells. These MMPs significantly increased in the control group compared with the normal group. In particular, MMP-1 decreased in a concentration-dependent manner compared with the control group, with IC_50_ value of 58.7 μM. In addition, the IC_50_ value of pseudoshikonin I on MMP-2 and MMP-13 was 60.8 and 63.3 μM, respectively. At a concentration of 100 μM, the inhibition effect of PSI on MMP-13 was 34.0% and 18.7%, respectively.

### 2.3. Effect of Pseudoshikonin I on the mRNA Expression of MMPs in IL-1β-Induced SW1353 Cells

**Figure 3:**
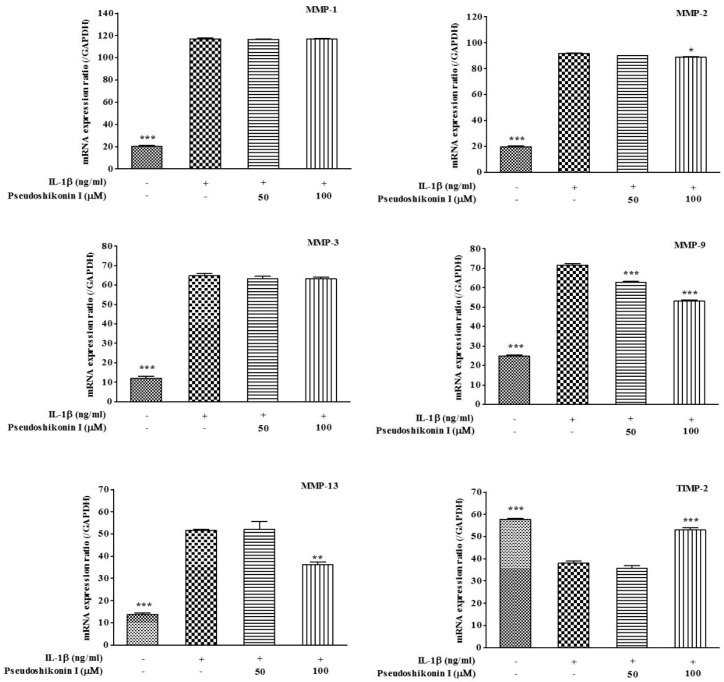
Effect of pseudoshikonin I on the gene expression of MMP-1, 2, 3, 9, 13, and TIMP-2 in IL-1β-induced SW1353 cells by RT-PCR. SW1353 cells incubated in the presence of 50 or 100 μM pseudoshikonin I stimulated with 20 ng/mL of IL-1β for 24 h. GAPDH was used as internal control. Values were expressed as means ± S.D. of triple experiments. * *p* < 0.05, ** *p* < 0.01, *** *p* < 0.001 compared with IL-1β treated group (control).

To assay the effect of pseudoshikonin I treatment on MMP-1, -2, -3, -9, -13, and TIMP-2 expression in IL-1β-stimulated SW1353 cells, cells were pretreated with pseudoshikonin I for 30 min alone or further treated with IL-1β (20 ng/mL) for 24 h. The gene expression of MMPs in SW1353 cells was investigated by RT-PCR (Table 2). AS shown in Figure 3, the relative MMP-1, -2, -3 mRNA expression showed no significant difference in the pseudoshikonin I (50 or 100 μM) treatment group; however, mRNA expression of MMP-9 (74.2% ± 1.4%), MMP-13 (70.2% ± 3.9%), iNOS (74.7%± 2.6%), and COX-2 (85.9% ± 3.2%) were suppressed by pseudoshikonin I treatment in a dose-dependent manner. In addition, the TIMP-2 mRNA expression was up-regulated by pseudoshikonin I (141.9 ± 0.6) at 100 μΜ treatment, compared to the IL-1β treated group (control, 100), and showed statistical significance (*p* < 0.001).

MMPs are enzymes that are important in the maintenance of the extracellular matrix. They assist in the creation of interstitial spaces by degrading extracellular matrix proteins, thereby facilitating multiple inflammatory processes [23]. MMP-1 (collagenase 1), the most abundant member of the MMP family, efficiently cleaves type II collagen in the cartilage and gets primarily synthesized by chondrocytes or fibroblasts in the connective tissues. MMP 13 (collagenase-3) is capable of cleaving aggrecans and type II collagen in the cartilage. Selective MMP-13 inhibitors are reported to be capable of blocking collagen degradation in cartilage explants efficiently without any musculoskeletal side effects [24,25]. MMP-3 (Stromelysin-1) cleaves proteoglycans, collagens, gelatins, and links proteins of aggrecan. Moreover, MMP-2 (gelatinase A) and MMP-9 (gelatinase B) are thought to play a key role in the degradation of type IV collagen and gelatin, the two main components of ECM [26]. It was proved that the expression of both MMP-2 and MMP-9 is enhanced in osteoarthritic cartilage. Therefore, the search of MMP inhibitor that modifies the expression and/or activity of MMPs might be considered a promising target for the treatment of OA.

## 3. Experiment

### 3.1. General

Multiple preparative liquid chromatography (MPLC, YMC LC-Forte/R, Kyoto, Japan) methods were performed on a column packed with reverse phase silica (RP-18). Kieselgel 60 F_254_ (Merck, Palo Alto, CA, USA) and RP-18 F_254S_ (Merck) were used as solid phases for the TLC experiment. The detection of spots on the TLC plate was performed by observing under a UV lamp (Spectronics Corp., New York, NY, USA) or spraying 10% aqueous H_2_SO_4_ on the developed plate followed by heating. Ultraviolet spectra were measured with a Shimadzu model UV-1601 spectrophotometer (Kyoto, Japan). QTOF-MS analysis was performed using a Waters Xevo G2-S QTOF MS (Waters Corp., Milford, MA, USA) operating in the negative ion mode. The NMR spectra were recorded on a Varian Inova AS 400 spectrometer (400 MHz, Varian, Palo Alto, CA, USA).

### 3.2. Plant Materials

Dried one year-old Lithospermi radix (LR) was purchased from Jacheon, Chungbuk Province, South Korea. A voucher specimen (MPS000071) was deposited at the Herbarium of the Department of Herbal Crop Research, National Institute of Horticultural and Herbal Science, Rural Development Administration, Eumseong, South Korea.

### 3.3. Extraction and Isolation

The powder of LR (1.5 kg) was extracted with 70% EtOH (15 L) at 80 °C for 2.5 h using a natural substance extractor (EG-BE1, Siheung, Korea) to obtain 70% EtOH extract. The EtOH extract was concentrated under vacuum using a rotary evaporator (N-1200B, EYELA, Tokyo, Japan) and dried in a freeze dryer (LP20, IlshinBioBase, Dongduchun, Korea) to yield the final test samples (LES, 424 g). The glass column (tayperling type, 50 mm × 150 mm, Dychrome, Sunnyvale, CA, USA) was pre-fitted with a glass guard column (15 mm × 25 mm) and an automatic fraction collector. The column was fitted with MPLC. Both of the columns were packed with RP-18 material (40–63 um). The pump was operated at pressure of 10–15 psi, and the column was preconditioned with acetonitrile/water (75:25) for 1 h. at flow rate of 10 mL/min to ensure that no other contaminants were present on the column. Crude extracts (4 g) were dissolved in acetonitrile/water (75:25, *v*/*v*, 10 mL) and loaded onto the guard column. Among the various solvent systems tried as eluents, acetonitrile/water (75:25) at flow rate of 10 mL·min^−1^ provided nigh teen fractions (F1-F19). Fraction 14 (150 mg) was further separately applied to the MPLC system and eluted with acetonitrile/water (80:20) at flow rate of 5 mL·min^−1^, yielding compound **1** (LE14-6, 28 mg).

Pseudoshikonin I (**1**). Reddish powder, [α]${}_{D}^{25}$ +75.0° (*c* = 0.10, CHCl_3_); negative QTOF/MS *m*/*z* 339.12884 [M − H]^−^; ^1^H-NMR (400 MHz, CD_3_OD, δ_H_) 7.42 (1H, s, H-2), 7.14 (1H, d, *J* = 3.2 Hz, H-8), 6.95 (1H, s, H-4), 6.69 (1H, d, *J* = 8.8 Hz, H-5), 6.54 (1H, dd, *J* = 8.8, 3.2 Hz, H-6), 5.75 (1H, dd, *J* = 6.8, 6.8 Hz, H-11), 5.69 (1H, m, H-2′), 5.11 (1H, m, H-13), 2.55 (2H, m, H-12), 2.14 (3H, s, H-5′), 1.89 (3H, s, H-4′), 1.74 (3H, s, H-16), 1.56 (3H, s, H-15); ^13^C-NMR (100 MHz, CD_3_OD, δ_c_) 167.5 (C-1'), 158.5 (C-1), 152.6 (C-7), 151.0 (C-9), 147.9 (C-10), 139.3 (C-2′), 135.6 (C-3′), 128.4 (C-3), 120.2 (C-2), 119.5 (C-14), 117.6 (C-8), 117.0 (C-6), 115.9 (C-4), 112.5 (C-5), 109.7 (C-13), 69.3 (C-11), 34.8 (C-12), 27.4 (C-5′), 25.9 (C-4′), 20.3 (C-15), 18.0 (C-16).

### 3.4. Cell Culture

Human SW1353 chondrosarcoma cells (American Type Culture Collection, Rockville, MD, USA) were cultured in Dulbecco’s modified Eagle’s medium (DMEM) containing 100 units/mL penicillin, 100 μg/mL streptomycin, and 10% fetal bovine serum (FBS; Gibco^®^, Grand Island, NY, USA) in six-well plates at 37 °C with 5% CO_2_. For the experiments, SW1353 cells were seeded in a six-well plate at 1 × 10^6^ cells/well. At confluence, cells were rinsed with PBS (pH 7.4, Gibco^®^) twice and treated with or without IL-1β at 20 ng/mL in the absence or presence of pseudoshikonin I for 24 h.

### 3.5. Determination of Cell Viability

Cell viability following treatment with PSI was determined by the 3′-(4,5,-dimethylthiazole-2yl)-2,5-diphenyl-tetrazolium bromide (MTT) assay. To examine the cytotoxicity of Pseudoshikonin I on SW1353 cells, the cells (1 × 10^5^ cells/well) were seeded in triplicate in 96-well plates and cultured in DMEM medium for 24 h. After pseudoshikonin I treatment, medium was removed from the plate and rinsed with PBS twice, 120 μL MTS solution (Promega, Madison, WI, USA) was added, and the plate was incubated at 37 °C with 5% CO_2_ for 2 h. Optical density (O.D.) was measured at 540 nm using a spectrophotometer (Multiskan™ Go Microplate Spectrophotometer, Thermo scientific, Foster City, CA, USA). Cell viability was calculated relative to untreated control cells as follows: (viability (% control) = 100 × (absorbance of treated sample)/(absorbance of control).

### 3.6. Measurement of MMPs Production in IL-1β-Induced SW1353 Cells

Following treatment with pseudoshikonin I, active MMP-1, -2, -3, -9, -13 and TIMP-2 were quantified in the cell culture supernatants using a commercially available ELISA kit (SensoLyte^®^ 520 MMPs assay kit, AnaSpec, Fremont, CA, USA) according to the manufacturer’s instructions. The fluorescence of 5-FAM can be read and monitored at excitation/emission wavelengths of 490/520 nm.

### 3.7. mRNA Isolation and RT-PCR in IL-1β-Induced SW1353 Cells

Following treatment with PS, chondrocytes were lysed and total cellular RNA was extracted with TRIZOL™ reagent (Invitrogen Life Technologies, Rockville, MD, USA) according to the manufacturer’s instructions. RNA integrity was assessed by electrophoresis on a denaturing 1.5% agarose gel. Total RNA (1 μg) was converted to cDNA using Maxime RT PreMix Oligo dT for RT-PCR (IntronBio, Seongnam, Korea). PCR was performed with incubation at 94 °C for 30 s, 57 °C for 45 s, and 72 °C for 45 s, with the final incubation at 72 °C for 7 min, with a Maxime PCR PreMix i-StarTaq (IntronBio, Seongnam, Korea). Gene-specific primers (Bioneer, Deajeon, Korea) used for the amplification of gene fragments are described in Table 1. The finished cDNA products were stored in aliquots at −80 °C until required. The RT-PCR products were electrophoresed in a 2% agarose gel and visualized by ethidium bromide (EtBr) staining. The relative expression of mRNA was normalized as the ratio to the expression of GAPDH mRNA, as a reference gene. All quantities were expressed as n-fold relative to a calibrator.

### 3.8. Statistical Analysis

All results were analyzed by GraphPad Prism^®^ Version 4.0 (GraphPad Software, San Diego, CA, USA) program for statistical analysis. Data are presented as means ± standard deviation (S.D.). Statistical significance was set to *p* < 0.05 and analysis of one-way ANOVA with Tukey’s post hoc comparisons.

## 4. Conclusions

In conclusion, this study shown that pseudoshikonin I (**1**) was isolated from Lithospermi radix. We have been generally successful in our attempt to the protective effects of pseudoshikonin I against IL-1β-induced MMPs production and mRNA expression in vitro using human chondrosarcoma cells. Pseudoshikonin I revealed any effect on the chondrocyte morphology and viability, or any significant cell death when compared with the control. This suggests that pseudoshikonin I was able to enhance the protective response of the matrix substrates in human chondrocytes and, possibly, had a favorable chondro-protective effect. However, the precise mechanism of pseudoshikonin I in relation of matrix degradation still needs to be clarified. Therefore, the action mechanism of pseudoshikonin I should be investigated in further studies.

## Figures and Tables

**Table 1:** IC_50_ value of pseudoshikonin I on the activity of MMP-1, 2, 3, 9, and 13 in IL-1β-induced SW1353 cells ^a^.

Matrix Metalloproteinase (MMP)	IC_50_ Value (μM)
MMP-1	58.7
MMP-2	60.8
MMP-3	>100
MMP-9	>100
MMP-13	63.3
TIMP-2	–

^a^ SW1353 cells incubated in the presence of 10, 25, 50, or 100 μM pseudoshikonin I stimulated with 20 ng/mL of IL-1β for 24 h. Results are expressed as the means ± S.D. of triple experiments.

**Table 2:** Primer sequences for reverse transcription PCR.

Gene Code	Accession ID	Sequence (5′→3′)		Temperature (°C)	Product Length (bp)
MMP-1	NM_001145938.1	Forward	AGTGACTGGGAAACCAGATGA	57	159
		Reverse	CGTCTTGGCAAATCTGGCCTGTAA		
MMP-2	NM_001127891.2	Forward	GCAGTGGGGGCTTAAGAAGA	57	969
		Reverse	AGCCGTACTTGCCATCCTTC		
MMP-3	NM_002422.3	Forward	ATTCCATGGAGCCAGGCTTTC	57	142
		Reverse	CATTTGGGTCAAACTCCACTGTG		
MMP-9	NM_004994.2	Forward	CATCCGGCACCTCTATGGTC	57	637
		Reverse	CATCGTCCACCGGACTCAAA		
MMP-13	NM_002427.3	Forward	AAATTATGGAGGAGATGCCCATT	57	125
		Reverse	TCCTTGGAGTGGTCAAGACCTAA		
TIMP-2	NM_003255.4	Forward	GTAGTGATCAGGGCCAAAGC	57	160
		Reverse	GGGGGCCGTGATAAACT		
GAPDH	NM_001256799.2	Forward	AGAAGGCTGGGGCTCATTTG	52	271
		Reverse	AGGGGCCATCAGTCTTC		

## References

[1] Cho M.H., Paik Y.S., Hahn T.R. (1999). Physical stability of shikonin derivatives from the roots of *Lithospermm erythrorhizon* cultivated in Korea. J. Agric. Food Chem..

[2] Ahn Y.H., Jin Y.H., Choe C.Y., Lee M.K., Lee S. (2009). Ecological characteristics of *Lithospermum erythrorhizon* population in habitats. Korean J. Physiol. Pharmacol..

[3] Rajasekar S., Park D.J., Park C., Park S., Park Y.H., Kim S.T., Choi Y.H., Choi Y.W. (2012). In vitro and in vivo anticancer effects of *Lithospermum erythrorhizon* extract on B16F10 murinemelanoma. J. Ethnopharmcol..

[4] Andújar I., Recio M.C., Giner R.M., Ríos J.L. (2013). Traditional Chinese medicine remedy to jury: The pharmacological basis for the use of shikonin as an anticancer therapy. Curr. Med. Chem..

[5] Pan Y.M., Liang Y., Wang H.S., Liang M. (2004). Antioxidant activities of several Chinese medicine herbs. Food Chem..

[6] Weng X.C., Xiang G.Q., Jiang A.L., Liu Y.P., Wu L.L., Dong X.W., Duan S. (2000). Antioxidant properties of components extracted from puccoon (*Lithospermum erythrorhizon* Sieb. et Zucc.). Food Chem..

[7] Han J., Weng X., Bi K. (2008). Antioxidants from a Chinese medicinal herb-*Lithospermum erythrorhizon*. Food Chem..

[8] Kim G.S., Park C.G., Lee K.H., Choi J.H., Lee S.E., Noh H.J., Lee J.H., Kim S.Y. (2011). Investigatin of shikonin pigments and antioxidant activity of the roots from *Lithospermum erythrorhizon* according to the different growth stages and areas of cultivation. Korean J. Med. Crop Sci..

[9] Chung H.S., Kang M., Cho C., Park S., Kim H., Yoon Y.S., Kang J., Shin M.K., Hong M.C., Bae H. (2005). Inhibition of lipopolysaccharide and interferon-gamma-induced expression of inducible nitric oxide synthase and tumor necrosis factor-alpha by Lithospermi radix in mouse peritoneal macrophages. J. Ethnopharmacol..

[10] Staniforth V., Wang S.Y., Shyur L.F., Yang N.S. (2004). Shikonins, phytocompounds from *Lithospermum erythrorhizon*, inhibit the transcriptional activation of human TNF-α promoter *in vivo*. J. Biol. Chem..

[11] Li M.Y., Xu Z.T., Zhu C.L., Wang J. (2012). Effect of different derivatives of shikonin from *Lithospermum erythrorhizon* against the pathogenic dental bacteria. Curr. Pharm. Anal..

[12] Sasaki K., Abe H., Yoshizaki F. (2002). *In vitro* antifungal activity of naphthoquinone derivatives. Biol. Pharm. Bull..

[13] Lee H.H., Yoon J.S., Song S.Y. (2010). Protective effect of *Lithospermum erythrorhizon* on galactosamine induced liver injury. Korean J. Microsc..

[14] Wang L., Li Z., Zhang X., Wang S., Zhu C., Miao J., Chen L., Cui L., Qiao H. (2014). Protective effect of shikonin in experimental ischemic stroke: Attenuated TLR4, p-p38MAPK, NF-κB, TNF-α and MMP-9 expression, up-regulated claudin-5 expression, ameliorated BBB permeability. Neurochem. Res..

[15] Kim J.S., Seo Y.C., No R.H., Lee H.Y. (2015). Improved cosmetic activity by optimizing the *Lithospermum erythrorhizon* extraction process. Cytotechnology.

[16] Hisamichi S.J., Yoshizaki F. (1982). Structures of new minor pigments and isolation of two isomers of shikonin derivatives from *Lithospermum erythrorhizon* Sieb. et Zucc.. Shoyakugaku Zasshi.

[17] Kim G.S., Kim H.J., Lee D.Y., Choi S.M., Lee S.E., Noh H.J., Choi J.G., Choi S.I. (2013). Effects of supercritical fluid extract, shikonin and acetylshikonin from *Lithospermum erythrorhizon* on chondrocytes and MIA-induced osteoarthritis in rats. Korean J. Med. Crop Sci..

[18] Zhou W., Jiang H.D.G.L., Peng Y., Li S.S. (2011). Comparative study on enantiomeric excess of main akannin/shikonin derivatives isolated from the roots of three endemic Boraginaceae plants in China. Biomed. Chromatogr..

[19] Zhang J.G., Lu Q., Wen H.D., Cai J.C. (2005). New asymmetric synthesis of alkannin and shikonin. Chin. Chem. Lett..

[20] Gebauer M., Saas J., Sohler F., Haag J., Söder S., Pieper M., Bartnik E., Beninga J., Zimmer R., Aigner T. (2005). Comparison of the chondrosarcoma cell line SW1353 with primary human adult articular chondrocytes with regard to their gene expression profile and reactivity to IL-1β^1^. Osteoarthr. Cartil..

[21] Tetlow L.C., Adlam D.J., Woolley D.E. (2001). Matrix metalloproteinase and proinflammatory cytokine production by chondrocytes of human osteoarthritic cartilage: Associations with degenerative changes. Arthritis Rheum..

[22] Kobayashi M., Squires G.R., Mousa A., Tanzer M., Zukor D.J., Antoniou J., Feige U., Poole A.R. (2005). Role of interleukin-1 and tumor necrosis factor α in matrix degradation of human osteoarthritic cartilage. Arthritis Rheum..

[23] Troeberg L., Nagase H. (2012). Proteases involved in cartilage matrix degradation in osteoarthritis. Biochim. Biophys. Acta..

[24] Burrage P.S., Mix K.S., Brinckerhoff C.E. (2006). Matrix metalloproteinases: Role in arthritis. Front. Biosci..

[25] Piecha D., Weik J., Kheil H., Becher G., Timmermann A., Jaworski A., Burger M., Hofmann M.W. (2010). Novel selective MMP-13 inhibitors reduce collagen degradation in bovine articular and human osteoarthritis cartilage explants. Inflamm. Res..

[26] Lipari L., Gerbino A. (2013). Expression of gelatinases (MMP-2, MMP-9) in human articular cartilage. Int. J. Immunopathol. Pharmacol..

